# Carboxymethyl Scleroglucan Synthesized via *O*-Alkylation Reaction with Different Degrees of Substitution: Rheology and Thermal Stability

**DOI:** 10.3390/polym16020207

**Published:** 2024-01-10

**Authors:** Rubén H. Castro, Isidro Burgos, Laura M. Corredor, Sebastián Llanos, Camilo A. Franco, Farid B. Cortés, Arnold R. Romero Bohórquez

**Affiliations:** 1 Grupo de Investigación en Fenómenos de Superficie—Michael Polanyi, Facultad de Minas, Universidad Nacional de Colombia—Sede Medellín, Medellín 050034, Colombia; caafrancoar@unal.edu.co (C.A.F.); fbcortes@unal.edu.co (F.B.C.); 2 Grupo de Investigación en Química Estructural (GIQUE), Escuela de Química, Universidad Industrial de Santander, Bucaramanga 680002, Colombia; isidroburgosortiz@gmail.com (I.B.); sllanosg@unal.edu.co (S.L.); arafrom@uis.edu.co (A.R.R.B.); 3 Centro de Innovación y Tecnología—ICP, Ecopetrol S.A., Piedecuesta 681011, Colombia; laura.corredor@ecopetrol.com.co

**Keywords:** scleroglucan, biopolymer, carboxymethylation, carboxymethyl scleroglucan, EOR

## Abstract

This paper presents the methodology for synthesizing and characterizing two carboxymethyl EOR-grade Scleroglucans (CMS-A and CMS-B). An *O*-Alkylation reaction was used to insert a hydrophilic group (monochloroacetic acid—MCAA) into the biopolymer’s anhydroglucose subunits (AGUs). The effect of the degree of the carboxymethyl substitution on the rheology and thermal stability of the Scleroglucan (SG) was also evaluated. Simultaneous thermal analysis (STA/TGA-DSC), differential scanning calorimetry (DSC), X-ray Diffraction (XRD), Fourier transform infrared (FTIR) spectroscopy, Scanning Electron Microscopy, and Energy Dispersive Spectroscopy (SEM/EDS) were employed to characterize both CMS products. FTIR analysis revealed characteristic peaks corresponding to the carboxymethyl functional groups, confirming the modification. Also, SEM analysis provided insights into the structural changes in the polysaccharide after the *O*-Alkylation reaction. TGA results showed that the carboxymethylation of SG lowered its dehydroxylation temperature but increased its thermal stability above 300 °C. The CMS products and SG exhibited a pseudoplastic behavior; however, lower shear viscosities and relaxation times were observed for the CMS products due to the breakage of the SG triple helix for the chemical modification. Despite the viscosity results, the modified Scleroglucans are promising candidates for developing new engineering materials for EOR processes.

## 1. Introduction

Enhanced oil recovery (EOR) processes aim to increase the oil recovery factor through thermal and chemical methods, which can alter fluid–fluid and rock–fluid interactions such as reduction of oil–water interfacial tension, oil swelling, oil viscosity reduction, or wettability alteration [1]. Polymer flooding consists of injecting a high molecular weight water-soluble polymer to improve the sweep efficiency in an oil reservoir by increasing the viscosity of the injected water [2]. The most commonly used synthetic polymer is partially hydrolyzed polyacrylamide (HPAM) [3]. However, HPAM solutions often suffer from viscosity loss due to chemical, mechanical, and thermal degradation [4], which affects their performance as an EOR additive [2]. Biopolymers such as Xanthan Gum [5], and Scleroglucan (SG) [6,7] have been proposed as an alternative to HPAM due to their excellent tolerance to mechanical shearing [8], high salinity [6], hardness, and high temperature [7]. Nevertheless, biopolymers are limited compared with HPAM due to their lower availability, higher production cost, and higher susceptibility to biodegradation.

SG is a natural non-ionic water-soluble polysaccharide obtained from fungal fermentation. Polysaccharides are formed by monosaccharides linked together by *O*-glycosidic bonds. The solubility, viscosity, surface, and interfacial properties of polysaccharides depend on the monosaccharide composition, types of bonding between monosaccharides, molecular structure (linear or branched), and molecular weight [9]. The unique chemical structure and higher molecular weight of SG provide distinctive physicochemical properties to this biopolymer (such as high-water solubility, pseudo-plasticity, resistance to hydrolysis, salt tolerance, and viscosity stability) [10], granting it an advantage over other polysaccharides.

The properties of polysaccharides can change depending on the producing strain and the production and downstream processing due to the modification of its molecular weight, the degree of polymerization and branching, conformational parameters, and purity [11]. The properties of polysaccharides can also be altered by introducing acidic, basic, or other functionality into their structures [12]. The modification methods can be selective and non-selective [13]. Non-selective methods introduce substituents randomly into the biopolymer chain and on each repeating unit. In contrast, the selective methods target specific functional groups.

In general, the modification methods of polysaccharides are classified into six types: sulfation [14], phosphorylation [15], carboxymethylation (an *O*-Alkylation reaction that uses a deprotonated alcohol and an organohalide to form an ether) [16], methylation [17], selenization [18], and acetylation [19]. The carboxymethylation of polysaccharides has been extensively investigated due to its simplicity. In the carboxymethylation, polysaccharides react with chloroacetic acid under basic conditions to replace the hydroxyl groups of polysaccharides with carboxymethyl groups, modifying their conformation, water solubility, and biological activity [18]. Different Carboxymethyl Scleroglucans have been synthesized and characterized for pharmaceutical applications [20,21,22]. For instance, Feeney et al. reported that the carboxymethylation prevents the triple helix formation of SG, and solutions of Carboxymethyl Scleroglucan at concentrations greater than 1% *w*/*v* exhibit a weak gel behavior [20]. Hydrophobic modifications of SG were performed by Nazmabadi et al. [23] and Bakhshi et al. [24] to obtain amphiphilic Scleroglucans for EOR processes. They reported that the hydrophobically modified Scleroglucans exhibited lower viscosity values and higher adsorption on oil-reservoir rock surfaces than SG.

In this study, SG was modified with monochloroacetic acid in the presence of sodium bicarbonate. The amount of sodium bicarbonate was changed to achieve two degrees of carboxymethyl substitution (DS). The effect of the DS on SG’s rheology and thermal stability was evaluated.

## 2. Materials and Methods

The chemicals used for the preparation of CMS-A and CMS-B were commercial EOR-grade Scleroglucans (SG, MW ~ 4.5 × 10^6^ Da, purity > 90%), monochloroacetic acid (MCAA, 99% pure, Sigma-Aldrich, St. Louis, MO, USA), sodium bicarbonate (NaHCO_3_, 99% pure, Sigma-Aldrich, St. Louis, MO, USA), and 2-propanol (CH_3_CH(OH)CH_3_, 99% pure, Sigma-Aldrich, St. Louis, MO, USA).

### 2.1. Carboxymethylation

**Figure 1:**
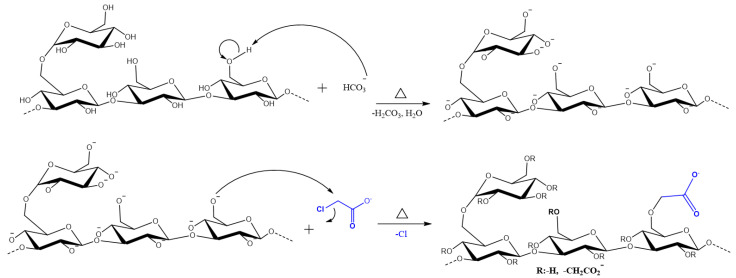
Scheme of the synthesis of Carboxymethyl Scleroglucan.

The carboxymethylation of SG is an *O*-Alkylation reaction, which consists of inserting a hydrophilic group (MCAA) in its anhydroglucose subunit (AGU). The synthesis scheme is reported in Figure 1 [25]. Only the substitution in the C6-position of the *D glucose* is shown, but it can occur in other positions (OH groups) [26].

For the reaction, 2 g of SG was added to 26.5 mL of 2-propanol, and the sample was stirred for 1 h at 700 rpm. To form the alkali groups, 0.125 g (for CMS-A) or 0.25 g (for CMS-B) of NaHCO_3_ was added to the solution. The sample was left to mix at 700 rpm at 25 °C for 30 min. Afterward, 1 g of MCAA was added and the reaction was carried out at 60 °C for 3.5 h under reflux, as recommended in previous studies [20,27]. Thereafter, the samples were washed three times with 2-propanol (3 × 26.5 mL) to remove the unreacted reagents. Finally, the precipitates were dried in a rotary evaporator under reduced pressure to yield 2.22 g and 2.48 g of CMA and CMB powder, respectively.

### 2.2. Characterization of CMS-A and CMS-B

The structural characterization of all the samples was carried out by FTIR using a Bruker Tensor 27 FTIR spectrophotometer (Alpha, Bruker, Billerica, MA, USA) with a total attenuated reflectance (ATR) platinum cell and analyzed using Bruker OPUS 7.5 software. Spectra were collected in the 4000–600 cm^−1^ range.

Thermal Analysis (STA/TG–DSC) was conducted using a Netzsch Simultaneous Thermal Analyzer (STA) 449 F5 Jupiter^®^. The curves were analyzed using the Netzsch Proteus-Thermal Analysis 6.1.0 software. The measurements were performed by heating 15 mg from 30 to 1000 °C at a heating rate of 5 °C/min under a nitrogen atmosphere.

The size and morphology of CMS-A and CMS-B were characterized through field emission gun scanning electron microscopy (FEG-SEM, QUANTA FEG 650 model, Thermo Fisher Scientific, Waltham, MA, USA) at ultra-high vacuum and an accelerating voltage of 25 kV. The samples were placed on metal stubs with carbon adhesive tape and coated with gold.

The elemental composition of both CMS products was determined with an energy-dispersive spectroscope (EDS) (EDAX Apolo X, Ametek, INC., Berwyn, PA, USA) with a resolution of 126.1 eV.

The X-ray Diffraction (XRD) pattern was recorded with a Bruker D-8 Advance A25 X-ray diffractometer (D8 Advance, Bruker, MA, USA) with CuKα Radiation at 40 kV voltage and 40 mA current to the X-ray generator. The scans were taken in the 2theta range from 2° to 70° with step-by-step scanning over 2theta angles. The samples were pressed between two glass slides on a flat sheet for the measurements.

#### Titration of CMS-A and CMS-B

The carboxymethyl group content, the number of carboxylic acids (-COOH), and SG’s degree of substitution (DS) were determined by reverse phase titration as recommended in previous studies [28,29,30,31]. For this, 1 g of SG was mixed with 50 mL of methanol, then 5 mL of 0.5 N aqueous solution of nitric acid was added, and the mixture was stirred for 10 min at room temperature. The sample was boiled for 5 min and stirred for 20 min at 300 rpm. After that, the sample was allowed to stand for 30 min. The sample was filtered and washed with ethanol (3 × 50 mL) to remove salts and acids. Afterward, the precipitate was washed with methanol (1 × 50 mL), transferred into a beaker, and heated until the alcohol evaporated. The CMS was dried in a conventional oven at 90 °C for 3 h. Then, 0.5 g of CMS was added into an Erlenmeyer flask with 100 mL of distilled water and 25 mL of 0.5 M aqueous solution of NaOH. The solution was heated at 98 °C. Finally, the heated solution was titrated with 0.3 M HCl using a phenolphthalein indicator until neutralization (when the color of the solution changed from magenta to transparent). The same procedure was carried out for a blank solution (0.5 M aqueous solution of NaOH without CMS). The carboxymethyl group [29] and -COOH content and the degree of substitution were calculated as follows:

-COOH content [29]:

(1) $$(\text{-}\mathrm{COOH})=(V_{o}-V_{n})\;\times \;N\;\times \;4$$

Carboxymethyl group content (%) [30]:

(2) $$(\%CM)=\frac{\left(V_{o}-V_{n}\right)\times 0.058\times 100\times N}{M}$$

Degree of substitution [30,32]:

(3) $$DS=\frac{162\times \%CM}{5800-(57\times \%CM)}$$

where $V_{n}$ is the volume of HCl used to neutralize the sample containing CMS (mL), $V_{o}$ is the volume of HCl used to neutralize the blank solution (mL), N is the normality of the HCl solution, 4 is a dilution factor, M is the amount of CMS used for the test (g), 162 is the molecular weight of anhydrous glucose unit, and 58 is the net increase in the anhydrous glucose unit for each substituted carboxymethyl group. Each titration was repeated at least three times to confirm the reproducibility.

### 2.3. SG and CMS Solutions Preparation

The CMS-A and CMS-B solutions at 1000 ppm were prepared as proposed by Abraham and Sumner [33] and Castro et al. [34]. For this, the SG, CMS-A, or CMS-B powder was dissolved into deionized water under mechanical stirring at 500 rpm. Then, the solutions were stirred at 800 rpm and heated at 40 °C for 10 min. Finally, the solutions were homogenized for 5 min using a high-performance immersion blender at 20,000 rpm (IKA™ T 25 Digital Ultra-Turrax, CPQ, Campinas, Brazil).

### 2.4. Viscosity of the SG, CMS-A and CMS-B Solutions

The viscosities of the fluids were measured in a DV_3_T viscometer (Brookfield Ametek, Middleborough, MA, USA) from 4.24 to 33.7 s^−1^ at 30, 60, and 80 °C. The uncertainties in viscosity results were 1% of the reported value. The viscosity data of SG, CMS-A, and CMS-B solutions exhibit a good fit [35] to the Carreau–Yasuda model [35,36].

## 3. Results

### 3.1. CMS-A and CMS-B Characterization

#### 3.1.1. ATR-FTIR Results

**Figure 2:**
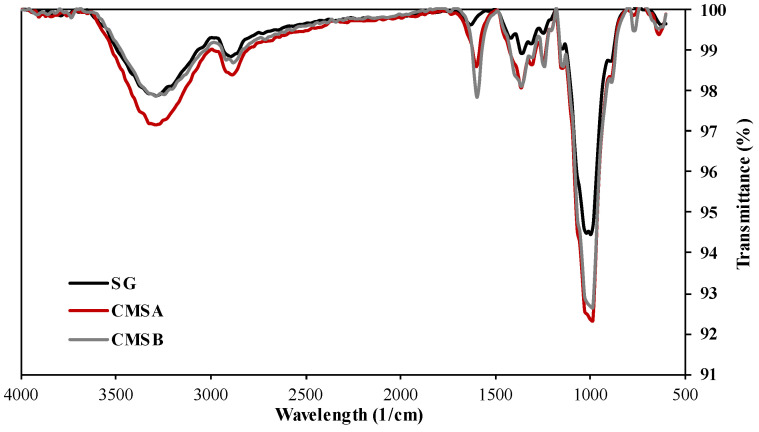
Infrared spectra of SG, CMS-A, and CMS-B.

The normalized FTIR spectra of SG, CMS-A, and CMS-B are shown in Figure 2. The adsorption band at 3200 cm^−1^ in both CMS products is attributed to the stretching vibration of their carboxyl groups and the SG’s free hydroxyl groups. The bands at around 2880 cm^−1^ and 1010 cm^−1^ correspond to the C-H stretching vibration and the -C-O-C- symmetrical bending vibrations, respectively. A low-intensity peak at 1600 cm^−1^ is observed in the SG spectra, which corresponds to the bending vibration of the OH groups. The intensity of this peak was increased by the chemical reaction due to the incorporation of the carboxylate groups (asymmetric stretching vibration) into the biopolymer chains. The intense peaks around 1400 cm^−1^ in both CMS correspond to the bending vibration of free secondary OH groups and the out-of-plane vibrations of C=O and OH groups of the carboxylic acid. According to Casadei et al. [21] and Corrente et al. [37], these peaks confirm the carboxymethylation of SG. On the other hand, Cerreto et al. [38] confirm the carboxymethyl groups’ presence using nuclear magnetic resonance (NMR) spectroscopy.

#### 3.1.2. STA/TG-DSC Results

**Figure 3:**
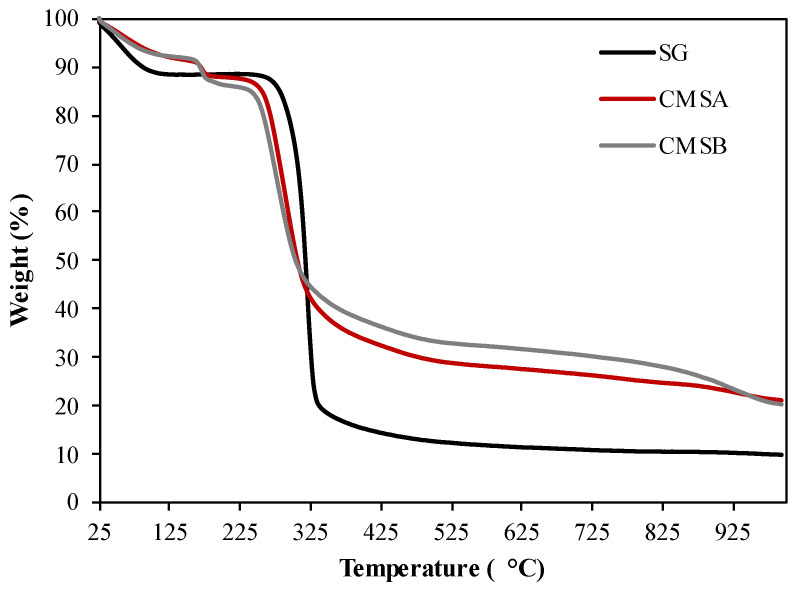
TG curves of SG, CMS-A, and CMS-B under nitrogen atmosphere.

Figure 3 shows the TGA profile of SG, CMS-A, and CMS-B. Three stages of weight loss were observed in the thermogram of SG. The first stage is observed below 100 °C corresponding to the reservoir temperature (DTG peak centered at 59.6 °C) and is attributed to the loss of the surface-adsorbed water. The weight loss in this zone was 11.02% (Table 1) [39]. The second stage was observed below 330 °C (DTG peak centered at 320.9 °C, Table 1) and is associated with the loss of structural water by dehydroxylation. The final stage (>330 °C) was assigned to the depolymerization reaction and the formation of volatile low molecular weight products [40].

On the other hand, four stages of mass loss were identified in the thermogram of both CMS products. The initial loss of surface-adsorbed water was observed as a DTG peak centered at 52 °C for CMS-A and at 52.9 °C for CMS-B (Table 1). The second mass loss stage, related to the decarboxylation of the carboxymethyl groups, occurred at 172.6 and 170.2 °C for CMS-A and CMS-B, respectively (Table 1) [41]. The last stages (T > 178 °C) were associated with the loss of structural water by dehydroxylation and biopolymer degradation.

The maximum weight loss rate (MWLR) parameters show that the depolymerization reactions are fastest in SG (13.35% min^−1^) and lowest in CMS-A (1.2% min^−1^) and CMS-B (2.42% min^−1^) in the nitrogen atmosphere (Table 1). This results in the lowest percent of char residue for SG and the highest in CMS-A [42].

**Figure 4:**
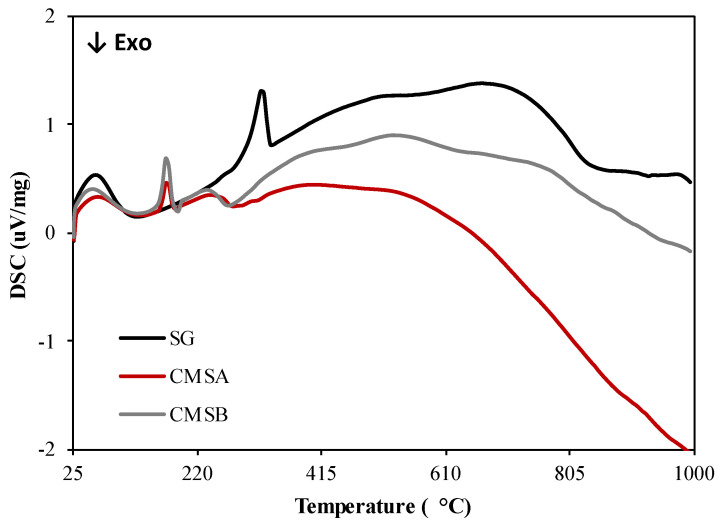
DSC curves of SG, CMS-A, and CMS-B under nitrogen atmosphere.

The DSC curves of all samples are presented in Figure 4. The decomposition of SG is an endothermic event with a peak maximum of 321.6 °C and occurs in the 304–330 °C temperature range. Also, the decomposition of CMS-A and CMS-B is an endothermic process that takes place in the temperature ranges 263–521 °C and 254–524 °C with peak maximum at 240 and 234.7 °C, respectively. The exothermic events in both CMS products at 279 °C could be attributed to the cleavage of the ether functional groups formed by the *O*-Alkylation. Based on the thermal analysis results, the carboxymethylation of SG lowered its dehydroxylation temperature [40] but increased its thermal stability above 300 °C.

#### 3.1.3. SEM-EDS Results

**Figure 5:**
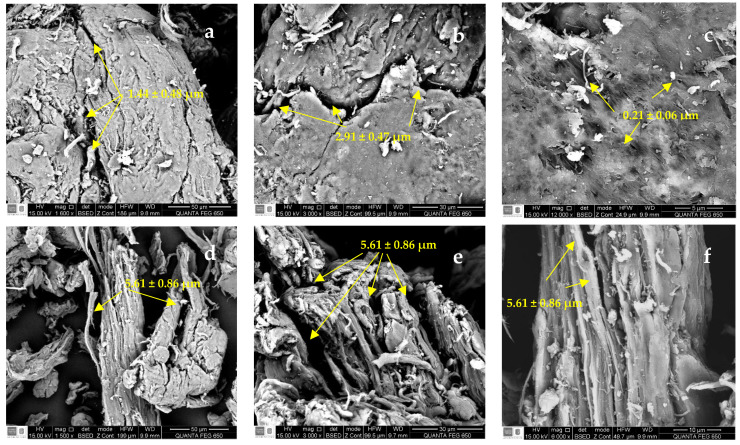

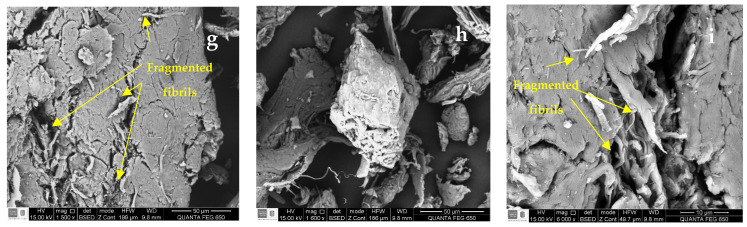
SEM micrographs of (**a**–**c**) SG at 1600×, 3000× and 12,000×, (**d**–**f**) CMS-A at 1600×, 3000×, 12,000×, and (**g**–**i**) CMS-B at 1600×, 3000×, 12,000×, respectively.

The SEM images of SG, CMS-A, and CMS-B are shown in Figure 5. SG has a fibrillar structure (Figure 5a–c) with openings between 1.44 ± 0.48 µm and 2.91 ± 0.47 µm and small cavities with an average diameter of 0.21 ± 0.06 µm. CMS-A (Figure 5d–f) has a less compact fibrillar structure with wider openings (5.61 ± 0.86 µm) than SG. The surface microstructure of CMS-B (Figure 5g–i) had no significant difference from SG’s. However, some disruptions in the network interconnections are observed as fragmented fibrils. The changes in the surface microstructure of SG confirm the functionalization of the biopolymer.

**Figure A1:**
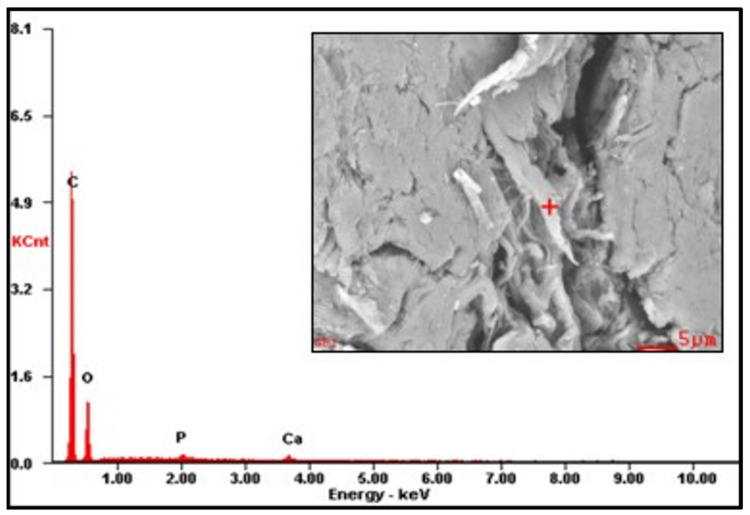
EDS spectrum of SG.

**Figure A2:**
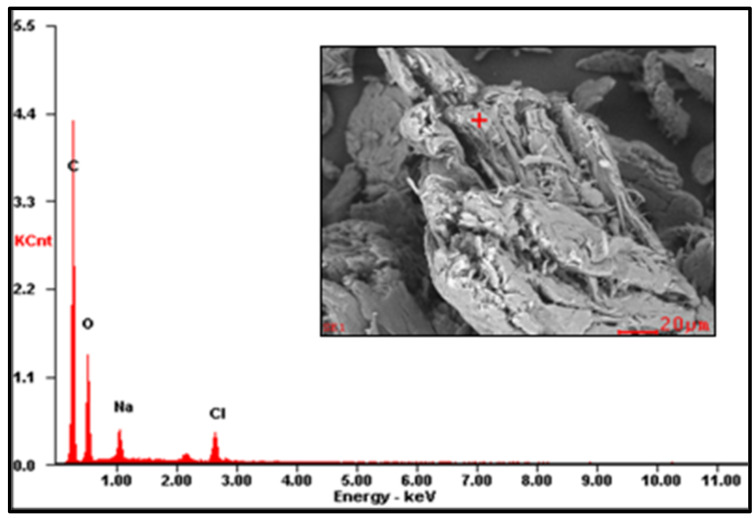
EDS spectrum of CMS-A.

**Figure A3:**
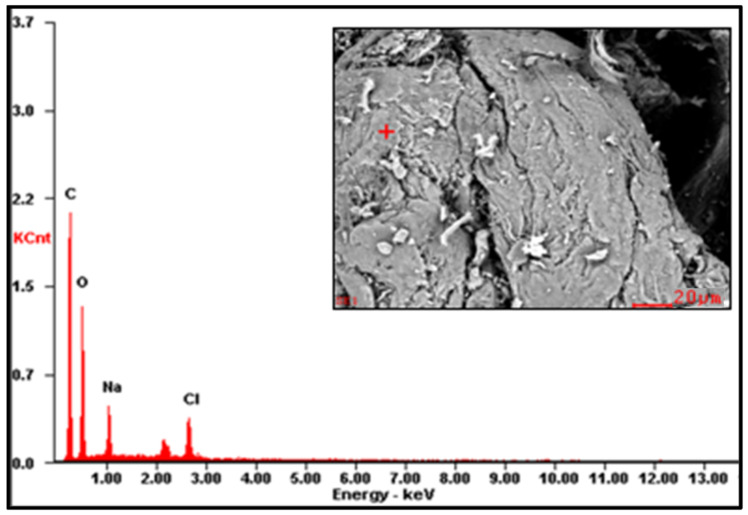
EDS spectrum of CMS-B.

The EDS results are reported in Table 2 (the EDS spectra are included in Appendix A, Figure A1, Figure A2 and Figure A3). The presence of Na on CMS-A and CMS-B confirms the carboxymethylation of the SG. However, the content of these elements on CMS-B is higher, suggesting that increasing the amount of NaHCO_3_ improves the reaction. In addition, the presence of Cl in both CMS products could be attributed to by-products of the reaction (i.e., salts).

The EDS results are confirmed with the calculation of the carboxymethyl group and the -COOH content and the degree of substitution (DS) (Table 3) because CMS-B exhibits the highest values. The DS of both products was optimal to synthesize SiO_2_/CMS nanohybrids that can retain the original viscosity of the SG during the microbial degradation bottle test performed by our research group. These research findings will be further reported.

#### 3.1.4. X-ray Diffraction (XRD)

**Figure 6:**
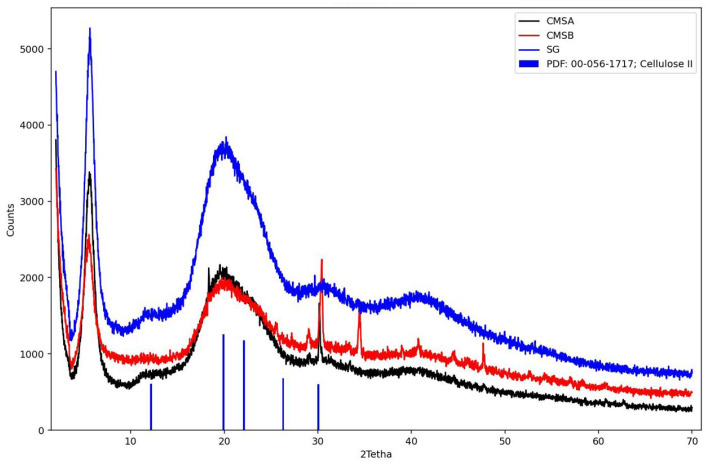
X-ray diffraction patterns of SG, CMS-A, CMS-B, and Cellulose II. PDF: Power diffraction file.

Figure 6 shows the diffractogram of SG, CMS-A, and CMS-B. These samples were compared with crystalline cellulose II. The characteristic peaks for cellulose were observed at 12.18°, 19.92°, 22.09°, 26.30°, and 30.07° [43]. In contrast, SG, CMS-A, and CMS-B exhibit a broad halo peak centered at a 2theta value of 20°, confirming the amorphous structure of these materials [44]. However, the peaks around 30° in the CMS-A and CMS-B curves show a partial ordering of the SG structure due to the *O*-Alkylation. CMS-B exhibits additional reflections around 34° and 48°, compared to CMS-A, which can be associated with their differences in the carboxymethyl group and the -COOH content, the degree of substitution, and a structural modification (Table 3).

### 3.2. Viscosity Measurements

**Figure 7:**
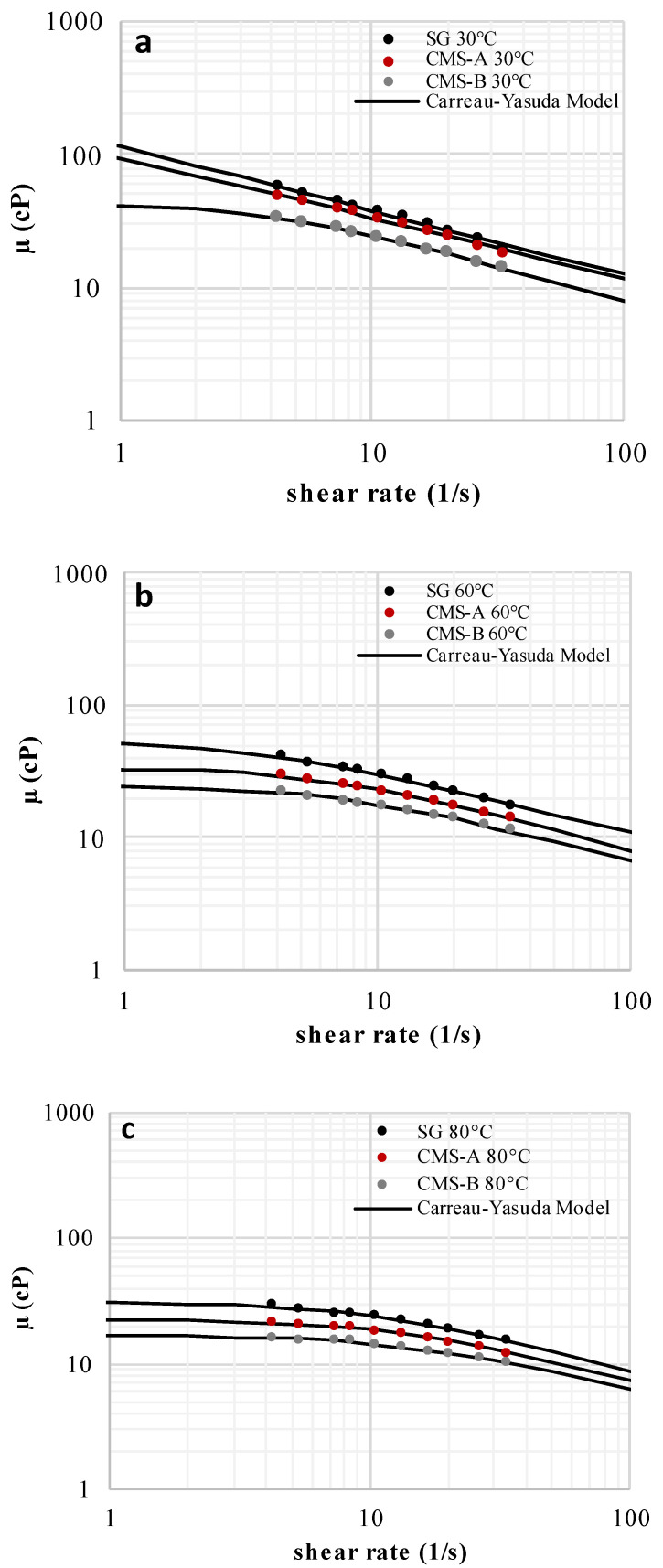
Flow curves of SG, CMS-A, and CMS-B at 1000 ppm and (**a**) 30 °C, (**b**) 60 °C, and (**c**) 80 °C [47].

The solutions of SG, CMS-A, and CMS-B exhibited a shear thinning behavior, which is attributed to the uncoiling and partial alignment of the biopolymer chains at the high shear rate region (Figure 7). The carboxymethylation reduced the SG viscosity (up to 8%) due to the breakage of its triple helix. In fact, at higher DS, lower viscosity values were obtained (DS CMS-B > DS CMS-A, µ CMS-B< µ CMS-A < µ SG). Despite these results, both CMS could be used for EOR processes because they have lower microbial degradation than EOR-grade Scleroglucan [45].

The Carreau–Yasuda model parameters of SG, CMS-A, and CMS-B are shown in Table 4. The zero-shear viscosity was reduced and the shear thinning behavior (n decreased) of the SG solutions intensified by increasing the DS because the *O*-Alkylation breaks the SG triple helix, facilitating the alignment of the SG chains at an applied shear rate. The relaxation parameter and the infinite shear viscosity were fixed at 1.5 s and 0.458 cP (water viscosity at 80 °C), respectively [46].

## 4. Conclusions

Two carboxymethyl derivatives of SG (CMS) with different degrees of substitution were synthesized through an *O*-Alkylation reaction by inserting a hydrophilic group (MCAA) in the anhydroglucose subunits (AGUs) of the biopolymer. XRD and FTIR confirmed the carboxymethylation of the SG, and the degree of substitution was determined by reverse phase titration. According to SEM-EDS analysis, SG and CMS-B have a similar surface microstructure. In contrast, CMS-A exhibits a less compact fibrillar structure than SG. Based on the thermal analysis results, the carboxymethylation of SG lowered its dehydroxylation temperature but increased its thermal stability below 130 °C and above 300 °C.

Finally, it can be concluded that the CMS with the highest DS (CMS-B) exhibits lower shear viscosity, zero-shear viscosity, and power-law exponent due to the breakage of the SG triple helix for the chemical modification, which facilitates the alignment of the biopolymer chains at an applied shear rate. Further studies will be performed to assess the impact of the degree of carboxymethylation on the thermal, chemical, mechanical, and microbial degradation of both CMS to determine their applicability as EOR additives.

## Figures and Tables

**Table 1:** TG and DTG parameters.

Sample	T (°C)	Weight Loss (%)	Endothermic Peaks of DSC (°C)	DTG Peaks ^a^ (°C)	MWLR ^b^ (% min^−1^)
SG	30–93	11.02	60.5	59.6	1.23
	304–330	67.57	321.6	320.9	13.35
	330–999	11.71	511.1		
	Residue, 1000	9.7			
CMS-A	30–99	7.58	62.9	52.0	0.80
	165–175	3.63	172.0	172.6	1.20
	263–521	59.35	240.0	290	4.31
	522–999	7.83	399.8		
	Residue, 1000	21.03			
CMS-B	30–87	7.91	54.5	52.9	0.86
	165–178	5.82	171.8	170.2	2.42
	254–524	52.87	234.7	276.3	3.86
	525–999	12.54	396.0		
	Residue, 1000	20.23			

^a^ First derivative of the TGA curve. ^b^ MWLR: maximum weight loss rate corresponding to the maximum peaks of DTG.

**Table 2:** Weight and atomic percentage of SG, CMS-A, and CMS-B by EDS analysis.

Sample	Element	Wt%	At%
	C	65.87	73.08
SG	O	30.89	25.73
	P	1.17	0.50
	Ca	2.08	0.69
	C	61.07	69.83
CMS-A	O	30.16	25.89
	Na	4.21	2.51
	Cl	4.57	1.77
	C	54.77	64.66
CMS-B	O	32.85	29.12
	Na	5.85	3.61
	Cl	6.52	2.61

**Table 3:** Carboxymethyl group content, -COOH content, and degree of substitution of SG, CMS-A, and CMS-B.

Sample	Method		
	Stojanovic [29]	Pushpamalar [30]	
	-COOH	%CM	DS
SG	0	0	0
CMS-A	0.57	7.36	0.22
CMS-B	0.91	13.63	0.44

**Table 4:** Carreau–Yasuda model parameters of SG, CMS-A, and CMS-B at 1000 ppm and 30 °C, 60 °C, and 80 °C.

Parameter	SG			CMS-A			CMS-B		
	30 °C	60 °C	80 °C	30 °C	60 °C	80 °C	30 °C	60 °C	80 °C
η_0_ (cP)	235.31	53.91	31.14	185.16	33.72	22.36	42.74	24.72	16.77
λ (s)	4.215	0.307	0.108	4.158	0.158	0.076	0.242	0.148	0.068
n	0.522	0.507	0.460	0.539	0.468	0.437	0.507	0.480	0.455

## Data Availability

The data presented in this study are available on request from the corresponding author.
